# Patterns of C1-Inhibitor Plasma Levels and Kinin–Kallikrein System Activation in Relation to COVID-19 Severity

**DOI:** 10.3390/life14121525

**Published:** 2024-11-21

**Authors:** Silvia Berra, Debora Parolin, Chiara Suffritti, Andrea Folcia, Andrea Zanichelli, Luca Gusso, Chiara Cogliati, Agostino Riva, Antonio Gidaro, Sonia Caccia

**Affiliations:** 1 Department of Biomedical and Clinical Sciences, Università degli Studi di Milano, 20157 Milan, Italy; berra.silvia@asst-fbf-sacco.it (S.B.); debora.parolin@unimi.it (D.P.); chiara.suffritti@policlinico.mi.it (C.S.); folcia.andrea@hsr.it (A.F.); luca.gusso@ospedaleniguarda.it (L.G.); chiara.cogliati@unimi.it (C.C.); agostino.riva@unimi.it (A.R.); 2 Department of Internal Medicine, Ospedale Fatebenefratelli, 20121 Milan, Italy; 3 Fondazione IRCCS Ca’ Granda Ospedale Maggiore Policlinico, Angelo Bianchi Bonomi Hemophilia and Thrombosis Center, 20122 Milan, Italy; 4 Division of Oncology, Unit of Urology, Urological Research Institute, IRCCS Ospedale San Raffaele, 20132 Milan, Italy; 5 Department of Biomedical Sciences for Health, Università degli Studi di Milano, 20133 Milan, Italy; andrea.zanichelli@unimi.it; 6 Operative Unit of Medicine, IRCCS Policlinico San Donato, San Donato Milanese, 20097 Milan, Italy; 7 Internal Medicine Unit, ASST Grande Ospedale Metropolitano Niguarda, 20162 Milan, Italy; 8 Department of Internal Medicine, Ospedale Luigi Sacco, 20157 Milan, Italy; 9 Department of Infectious Diseases, Ospedale Luigi Sacco, 20157 Milan, Italy

**Keywords:** C1-INH, C1-INH functional, kinin–kallikrein system (KKS), C1-INHIBITOR-kallikrein complexes, COVID-19

## Abstract

Background: Although more than four years have passed since the pandemic began, SARS-CoV-2 continues to be of concern. Therefore, research into the underlying mechanisms that contribute to the development of the disease, especially in more severe forms, remains a priority. Sustained activation of the complement (CS), contact (CAS), and fibrinolytic and kinin–kallikrein systems (KKS) has been shown to play a central role in the pathogenesis of the disease. Since the C1 esterase inhibitor (C1-INH) is a potent inhibitor of all these systems, its role in the disease has been investigated, but some issues remained unresolved. Methods: We evaluated the impact of C1-INH and KKS on disease progression in a cohort of 45 COVID-19 patients divided into groups according to disease severity. We measured plasma levels of total and functional C1-INH and its complexes with kallikrein (PKa), reflecting KKS activation and kallikrein spontaneous activity. Results: We observed increased total and functional plasma concentrations of C1-INH in COVID-19 patients. A direct correlation (positive Spearman’s r) was observed between C1-INH levels, especially functional C1-INH, and the severity of the disease. Moreover, a significant reduction in the ratio of functional over total C1-INH was evident in patients exhibiting mild to intermediate clinical severity but not in critically ill patients. Accordingly, activation of the KKS, assessed as an increase in PKa:C1-INH complexes, was explicitly observed in the mild categories. Conclusions: Our study’s findings on the consumption of C1-INH and the activation of the KKS in the less severe stages of COVID-19 but not in the critical stage suggest a potential role for C1-INH in containing disease severity. These results underscore the importance of C1-INH in the early phases of the disease and its potential implications in COVID-19 progression and/or long-term effects.

## 1. Introduction

A novel virus, severe acute respiratory syndrome coronavirus 2 (SARS-CoV-2), causing coronavirus disease (COVID-19), was first identified in December 2019 in Wuhan, China, and rapidly spread worldwide. On 11 March 2020, the World Health Organization (WHO) officially declared COVID-19 a global pandemic disease. The COVID-19 pandemic caused an enormous health and socio-economic impact on human society worldwide and, up to date, caused more than 7 million deaths [1]. Despite a decline in its virulence, SARS-CoV-2 continues to represent a significant threat to human health, even considering the persistence of long-term symptoms following acute infection. Factors such as age, sex, underlying health conditions, and the severity of the initial COVID-19 infection can contribute to the likelihood of developing these long-term symptoms [2]. Ongoing research and international cooperation continue to enhance our understanding of COVID-19, guiding global efforts to mitigate its impact and ensure a safer future.

The latest evidence suggests that the clinical picture of COVID-19 should be viewed as a thromboinflammatory disorder, encompassing both cellular inflammation and its intricate interplay with coagulopathy [3].

As a matter of fact, it has been established that plasma proteolytic cascades, including the complement (CS), coagulation, contact (CAS), fibrinolytic, and kinin/kallikrein systems (KKS), exert pivotal roles within SARS-CoV-2 infection [4,5,6,7,8,9,10,11,12]. In the context of the COVID-19 pathomechanism, these cascades activate a number of highly interconnected and often overlapping immunological and hemostatic processes, hence the term immunothrombotic cascades.

C1 esterase inhibitor (C1-INH) is an abundant plasma serpin (serine protease inhibitor) that facilitates the regulation of all these systems by the inhibition of several serine proteases, thereby contributing to homeostasis in the generation of proinflammatory and thrombotic mediators [13]. It owes its name to its function as the primary inhibitor of the CS, inactivating C1r and C1s proteases in the classical pathway and mannose-binding lectin-associated serine protease 1 and 2 (MASP-1/MASP-2) in the lectin pathway [14,15,16]. However, its pivotal role in preventing uncontrolled activation of the CAS and KKS pathways is gaining increasing recognition [17,18,19,20,21]. CAS and KKS interact closely in the intravascular compartment. The CAS consists of factor XII (FXII), which auto-activates on negatively charged surfaces to form FXIIa, which then reciprocally activates prekallikrein (PK) to form plasma kallikrein (PKa). The system is accelerated by the presence of the cofactor high molecular weight kininogen (HK). The CAS constitutes a component of the innate immune response, initiating both blood coagulation and inflammation when triggered by vascular injury. The KKS is a biochemical pathway that produces bradykinin (BK), a peptide that causes vasodilation, increased vascular permeability, and pain. It is involved in the regulation of blood pressure, inflammation, and coagulation. The key protease of the KKS is plasma kallikrein (PKa), which converts HK into BK. Indeed, the generation of PKa by FXIIa in the CAS provides a link between the two pathways. In this context, C1-INH serves to inhibit both FXIIa and PKa.

Moreover, C1-INH exerts minor inhibitory activity towards activated factor XI (FXIa) and thrombin of the coagulation system, as well as on plasmin and tissue-type plasminogen activator (tPA) of the fibrinolytic system [22,23,24].

Increased plasma levels of C1-INH in COVID-19 patients have been described in several studies [25,26,27,28,29]. The same was also confirmed at a transcriptomic level. Indeed, the *SERPING1* transcript, encoding for C1-INH, was found to be increased in the whole blood RNA of COVID-19 patients compared to healthy controls [25,30].

A bioinformatic study on interactomes suggests that SARS-CoV2 proteins can interact with C1-INH, thus sequestering it and preventing its action [31]. Russo et al. hypothesize that SARS-CoV2 proteases could cleave the N-terminal region of C1-INH, blocking its function and increasing inflammatory processes [32].

However, the suggestion that while C1-INH levels increase in COVID-19 patients, its functionality might be compromised has been proposed but has not been explored in depth.

Due to its role as a regulator of the plasma proteolytic cascades, in fact or potentially activated in COVID-19, C1-INH was identified as a promising treatment candidate. However, to date only a few clinical and preclinical studies have been performed, with not always concordant results and several uncertainties remaining as to the correct dose and treatment duration [33].

Clinical trials using recombinant C1-INH (rhC1-INH) (Conestat alfa, Pharming Group/Salix Pharmaceuticals) or plasma-derived C1-INH versus Icatibant (a bradykinin B2 receptor antagonist) have generally shown improved clinical outcomes, although results have not always been consistent [34,35,36,37,38,39,40].

In the present study, we aimed to better characterize the impact of C1-INH on COVID-19 disease status. Therefore, in addition to measuring C1-INH plasma levels, we also evaluated its function.

## 2. Materials and Methods

### 2.1. Materials

Berinert P (CSL Behring, King of Prussia, PA, USA) was dissolved in water as indicated by the manufacturer and used as standard C1-INH for ELISAs or to form complexes with kallikrein. Active human kallikrein (HPKa 1303, Enzymes Research Laboratories, South Bend, IN, USA) was used to form complexes with C1-INH or biotinylated to be used in the functional ELISA assay.

We used the following primary antibodies: an in-house polyclonal chicken anti-C1 INH antibody [41] unlabeled or biotinylated, goat anti-C1 INH antiserum (C8159, Merck, Darmstadt, Germany), sheep anti-human Prekallikrein (CL20090A Cedarlane, Burlington, ON, Canada), and anti-HK light chain (GAHu/HMWK—Nordic, Tilburg, The Netherlands). Secondary antibodies were from Merck: anti-goat IgG HRP (A5420), anti-chicken IgY AP (A9171), anti-sheep IgG AP (A5187), and biotinylated rabbit anti-goat antibody (B7014).

Streptavidin-poly-HRP (21140, Pierce) was from Thermo Fisher Scientific (Waltham, MA, USA), and Streptavidin (S4762) and high molecular weight (500 kDa) dextran sulfate (DXS) (D6001) were from Merck. TMB (3,3′,5,5′-Tetramethylbenzidine) peroxidase substrate (5120-0047) was from KPL (Gaithersburg, MD, USA).

The chromogenic peptide substrate H-D-Pro-Phe-Arg-pNA (S-2302) for kallikrein functional assays was from Chromogenix, Instrumentation Laboratory (Bedford, MA, USA).

Buffer formulation was as follows: Coating buffer (0.05 M carbonate-bicarbonate, pH 9.6); Hepes Binding buffer (20 mM HEPES pH 7.4, 150 mM NaCl, 0.5 mg/mL BSA); HBS sample buffer (10 mM HEPES pH 7.4, 150 mM NaCl, 1 mM MgSO_4_, 5 mM KCl, 0.1% (*v*/*v*) Tween-20 and cOmplete™ Protease Inhibitor Cocktail (04 693 124 001—Roche, Basel, Switzerland); and Tris/Phosphate buffer (10 mM sodium phosphate, 50 mM Tris, 150 mM M NaCl, 1% (*v*/*v*) Tween, pH 8.0).

For all the ELISA tests, Nunc^®^ Maxisorp^®^ 96-well microtiter plates (460984—Thermo Fisher Scientific, Waltham, MA, USA) were used.

Spectra Max 190 photometer and SoftMax^®^ Pro 7 software (Molecular Devices, Eugene, OR, USA) were used to read the plates and for quantification using a 4-parameter Logistic fitting.

### 2.2. Patient Selection and Definition

We enrolled a cohort of unvaccinated SARS-CoV-2-infected adult patients who received care for COVID-19 at Sacco Hospital in Milan. A total of 45 hospitalized patients were included in this study according to the following inclusion criteria: 1, COVID-19 disease confirmed by a positive SARS-CoV-2 RT-PCR test result from a nasopharyngeal swab specimen; 2, available sample for C1-inhibitor analysis taken when the patient was hospitalized due to acute SARS-CoV-2 infection; 3, accessible digital hospital record to extract clinical data; 4, age ≥18 years. The exclusion criteria were as follows: 1, previous vaccination with any COVID-19 vaccine, and 2, the diagnosis of hereditary angioedema (HAE) as a disorder affecting C1-INH.

The control group (CTR) consisted of 18 healthy subjects with no evidence of SARS-CoV-2 infection, matched by age, gender, and blood sampling time.

Ethical approval for this study was obtained from the ASST Fatebenefratelli-Sacco Ethics Committee (APPROVAL NUMBER 2020/ST/049).

Written informed consent was obtained from the patients or from the closest relative available if the patient was unable to give consent. The Declaration of Helsinki and its subsequent revisions were followed.

Patients were divided into groups according to WHO’s COVID-19 severity classification (Table 1) (https://www.who.int/publications/i/item/WHO-2019-nCoV-clinical-2023.1, accessed on 11 May 2024) [42].

Severity was first assessed during hospitalization and subsequently when sampling was performed. Peak in-hospital severity was also registered.

### 2.3. Blood Sampling and Laboratory Tests

The samples were obtained from blood withdrawn using either sodium citrate 3.2% or EDTA as an anticoagulant. After centrifugation at 2000× *g* for 20 min at room temperature, the plasma was divided into aliquots and stored at −80 °C until tested.

All routine laboratory tests, including standard clinical chemistry, inflammatory parameters, and complete blood counts, were performed at the hospital’s central laboratory.

### 2.4. Preparation of PKa:C1-INH Complexes

Complexes between kallikrein and C1-INH (PKa:C1-INH) were prepared as follows. Active human kallikrein (HPKa 1303) was incubated with a 12-fold molar excess of C1-INH for 90 min at 37 °C to ensure that all the PKa was complexed to C1-INH. The formation of complexes and the potential presence of remaining PKa were checked by SDS-PAGE. Moreover, the residual PKa activity was tested by a chromogenic assay using H-D-Pro-Phe-Arg-pNA (S-2302) as a substrate. Given the absence of free PKa in the SDS-PAGE and the negligible residual activity observed in the complex reaction mix, we assumed that all the PKa was complexed to C1-INH, and the concentration of the PKa:C1-INH complexes corresponded to that of the PKa used (1.8 μM). The PKa:C1-INH complexes obtained were used as a standard in the ELISA assay for the detection of circulating PKa:C1-INH complexes.

### 2.5. ELISAs

Total C1-INH plasma levels were measured with a homemade ELISA. Plates were coated with 75 ng/well of an in-house chicken anti-C1 INH antibody in coating buffer overnight at 4 °C. Plates were blocked with 3% BSA in TBS Tween at 37 °C for 1 h to avoid non-specific binding, and subsequently plasma samples and a serial dilution of standard C1-INH were applied at 37 °C for 1 h. To detect the signal, the plates were incubated with a goat anti-C1 INH antiserum (C8159) 1:2500 followed by an anti-Goat IgG HRP (A5420) 1:40,000. Finally, the reaction was developed by exposure to TMB peroxidase substrate stopped with 2 M sulfuric acid, and the absorbance was measured at 450 nm.

C1-INH inhibitory function against PKa was measured by an immunoenzymatic assay as described by Joseph [43] with minor modifications. Briefly, plates were coated with 1 μg/mL Streptavidin (S4762) in coating buffer overnight at 4 °C, and unused sites were blocked with 1.5% BSA in PBS at 37 °C for 1 h. Samples and standards were prepared in Hepes Binding buffer and added to the plates (50 μL each); 50 μL of biotinylated PKa (1 μg/mL) were added to all wells, and after mixing, the plates were incubated at 37 °C for 1 h. The signal was revealed with goat anti-C1 INH antiserum and anti-Goat IgG HRP as before.

To test assays consistency, assessment of total and functional C1-INH was also performed with commercially available assays in a group of patients: radial immunodiffusion (Human C1 Inactivator BINDARID—RN019.3, The Binding Site, Birmingham, UK) for total C1-INH and a chromogenic assay (Technocrom^®^ C1 inhibitor—5345003, Technoclone GmbH, Vienna, Austria) for C1-INH function.

Complexes between C1-INH and PKa were assessed with a home-made ELISA assay, operating major modifications from the already published methods [44,45]. For the quantification, linear dilutions of the standard of PKa:C1-INH complexes were used, allowing the expression of quantities as molar concentrations. Moreover, to obtain a value for plasma activation, a calibrator was produced as follows. Normal pooled plasma from healthy donors (NPP) was activated in vitro with high molecular weight (500 kDa) dextran sulfate (DXS) for 30 min at 37 °C and subsequently mixed with non-activated NPP to generate different percentages of activated plasma (5–10–20–30%).

Plates were coated with 500 ng/well of sheep anti-human Prekallikrein antibody (CL20090A) in coating buffer overnight at 4 °C and subsequently blocked with 3% skimmed milk in TBS Tween at 37 °C for 1 h. PKa:C1-INH complex standards, plasma samples (1:16), and calibrators (1:16) were diluted in HBS sample buffer, applied to the plate, and incubated at 37 °C for 1 h. The PKa:C1-INH complexes were detected with a biotinylated polyclonal chicken anti-C1 INH antibody followed by streptavidin-poly-HRP 1:20,000. Finally, the reaction was developed with TMB, stopped with 2 M sulfuric acid, and the absorbance at 450 nm was measured.

Values were first expressed as concentrations in nanomolar (nM) and subsequently transformed into percentages of activation using the calibrator.

### 2.6. Western Blot

Western Blot was employed to assess the presence of cleaved C1-INH or PKa:C1-INH complexes in patients’ plasma (antibodies and conditions used are detailed in Table 2).

Plasma samples were first diluted 1:10 (*v*/*v*) in PBS; subsequently, 5 μL were diluted 25% (*v*/*v*) in 4× Laemmli sample buffer, with the addition of 50 mM DTT only for the PKa:C1-INH complexes, and boiled for 5 min. Samples were subjected to SDS-PAGE on home-made Tris/glycine gels or on 4–15% Mini-PROTEAN TGX precast gels (4561083 Bio-Rad, Hercules, CA, USA) in TGS Buffer. Gels were blotted on a PVDF membrane using the Trans-Blot Turbo Blotting System with Trans-Blot Transfer Pack (1704156—Bio-Rad, Hercules, CA, USA). Membranes were blocked for 1 h with 3% skimmed milk in TBS Tween and subsequently incubated with specific antibodies (see Table 2). The signal was revealed after a 10 min incubation with SigmaFAST^TM^ BCIP/NBT (B5655, Merck).

The membranes were acquired with an Epson Perfection V800 Photo scanner (dual lens system, Epson, Suwa, Japan). Densitometric analysis was performed using ImageJ (ImageJ Software, version 1.47; Newark, CA, USA).

The percentage of cleaved C1-IHN was calculated by taking the ratio of the band corresponding to the cleaved form to the sum of the intensities of both bands (intact and cleaved), representing the total C1-IHN.

### 2.7. Amidolytic Activity of Kallikrein

The amidolytic activity of kallikrein was determined using a modification of a method based on the chromogenic peptide kallikrein substrate H-D-Pro-Phe-Arg-pNA (S-2302) [46].

Briefly, 150 μL of plasma diluted 1:45 in Tris/Phosphate buffer were loaded in triplicate in a microtiter plate. After the addition of 50 μL of 1 mM S-2302 substrate, the plate was immediately transferred into a microplate reader set at 37 °C. The release rate of paranitroaniline (pNA) was photometrically measured at 405 nm. The total kallikrein-like activity was obtained from the slope of the linear phase of the kinetic curve.

### 2.8. Statistics

Statistical analysis was performed with GraphPad Prism 8.4 (GraphPad Software, San Diego, CA, USA). Categorical data are reported as frequencies, expressed as percentages (%). The Kolmogorov–Smirnov test was performed to evaluate the normality of the data distributions. Given the non-normal distribution of the variables, the Kruskal-Wallis non-parametric statistical test was employed to ascertain differences between multiple independent groups. A Spearman rank correlation test was used to assess dependence between variables.

Statistical significance is represented with * if *p* < 0.05, ** if *p* < 0.01, *** if *p* < 0.001, and **** if *p* < 0.0001.

## 3. Results

### 3.1. Characteristics of the Study Population

Our study involved an adult cohort of 45 unvaccinated SARS-CoV-2-infected, hospitalized patients who were admitted at Luigi Sacco Hospital in Milan for COVID-19. The average age was 60 years. The median length of the hospitalization was 15 days. The lethality rate was 17%, with a median of 13 days between disease onset and death. The clinical characteristics of patients are indicated in Table 3.

Eighteen subjects with no evidence of SARS-CoV-2 infection, matched by age, gender and time of blood sampling, formed the healthy control group.

Clinical and treatment data indicating the severity of COVID-19 were extracted from electronic hospital records, and patients were divided into four severity categories according to World Health Organization (WHO) criteria for the clinical management of COVID-19 [42].

Hospitalized symptomatic patients without any evidence of viral pneumonia or hypoxia were classified as mild. Moderate and severe categories, respectively, represent patients with pneumonia who require low or medium-high flow oxygen therapy and non-invasive mechanical ventilation. Finally, the critical class is composed of patients with acute respiratory distress syndrome (ARDS) needing invasive ventilation.

Patients’ stratification and statistical analysis were performed at three different time points: first, when the patient was admitted for hospitalization; second, at the time of sample collection; and third, according to the worst clinical condition (or death).

The results of the statistical analysis refer to the WHO severity class relative to the day of withdrawal. No significant changes in the results were observed when the classification was based on the day of enrollment or the peak of the disease.

In line with other publications, we observed a positive correlation between WHO classes and C-reactive protein (CRP), interleukin-6 (IL-6), ferritin, D-dimers, and neutrophil count. Conversely, an inverse correlation (negative Spearman’s r) was observed with lymphocyte count [47,48,49,50]. No correlation was present with coagulation tests (Table 4).

### 3.2. Dysregulation of C1-INH in COVID-19

The plasma levels of total C1-INH were higher in COVID-19 patients compared to controls, with a statistically significant positive correlation (Spearman r = 0.58; *p* < 0.0001).

**Figure 1:**
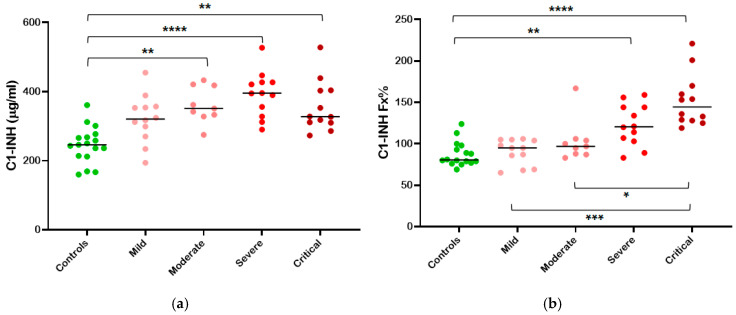
Plasma C1-INH evaluation in relation to the severity of COVID-19. Patients were divided into groups according to WHO severity classification (Table 1). Dot plots of plasma levels of total (**a**) and functional (**b**) C1-INH of healthy (green, n = 18), mild (light pink, n = 12), moderate (dark pink, n = 9), severe (red, n = 12), and critical (maroon, n = 12) individuals. Medians (horizontal bars) depict non-Gaussian data; *p* values are from the Mann–Whitney test. Statistical significance is represented with * if *p* < 0.05, ** if *p* < 0.01, *** if *p* < 0.001, and **** if *p* < 0.0001.

When all classes of clinical severity were considered, a positive trend of increase was observed from mild to severe disease. However, a trend reversal in the median concentration was evident for critically ill patients, although not statistically significant (327 μg/mL for critical vs. 395 μg/mL for severe) (Figure 1a).

Statistically significant differences were present between the control group and patients with moderate (*p* < 0.01), severe (*p* < 0.0001), and critical (*p* < 0.01) disease.

Although C1-INH is in excess compared to physiological conditions, it could be easily overwhelmed by the proteases activated in COVID-19. Therefore, to estimate C1-INH consumption, in addition to measuring the total C1-INH levels, we also assessed C1-INH functional levels as well as the presence of complexes between C1-INH and PKa.

Considering C1-INH functional levels, the rise of C1-INH with increasing clinical severity was confirmed (Spearman r = 0.7353; *p* < 0.0001), but differences emerged between patients with different scores. No more significant differences between controls and mild or moderate severity patients were observed, while the variations with higher severity classes remained evident (Figure 1b).

The data presented were obtained through ELISA quantification, and comparable results for total and functional C1-INH were achieved using commercially available assays.

To better understand the slight discrepancy observed between the increase in total and functional C1-INH levels, we decided to consider the fraction of functional over total C1-INH (Fx/tot). This fraction represents the portion of total C1-INH that is actually active as a protease inhibitor, allowing for a more accurate evaluation of C1-INH consumption.

**Figure 2:**
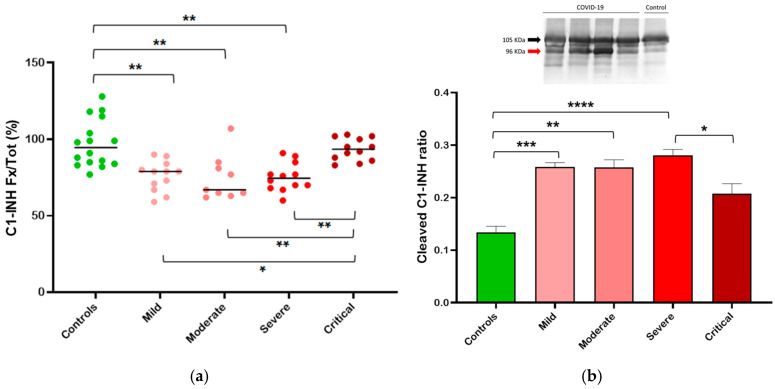
Consumption of plasma C1-INH in relation to the severity of COVID-19. (**a**) Dot plot showing the fraction of functional (Fx) over total (Tot) C1-INH; (**b**) bar graph indicating the ratio of cleaved C1-INH as determined from Western blot (a representative gel is shown in the insert). Median (horizontal bars) depict non-Gaussian data; *p* values are from the Mann–Whitney test. Statistical significance is represented with * if *p* < 0.05, ** if *p* < 0.01, *** if *p* < 0.001, and **** if *p* < 0.0001.

The fraction of functional over total C1-INH was significantly different between healthy controls and patients of the mild (*p* < 0.01), moderate (*p* < 0.01), and severe (*p* < 0.01) categories, while no differences were observed between controls and critically ill patients. At the same time, critically ill patients differed from patients of lesser clinical severity. It is evident that the mild-intermediate categories are characterized by a reduction in the C1-INH functional fraction, down to a median of 67% in the moderate class. Therefore, in mild-intermediate-level patients, a large amount of total C1-INH is inactive, suggesting that there is a consumption of C1-INH. On the other hand, in controls and critically ill patients, the fraction is approximately 100%, indicating that the majority of the C1-INH present is functionally active (Figure 2a).

These results were also confirmed by SDS-PAGE and Western blotting. Since functional protein migrates differently from cleaved and complexed (inactive) forms, it is possible to calculate the portion of active or inactive C1-INH by comparing the relative intensities of the different bands. As illustrated in the insert of Figure 2b, intact C1-INH migrates as 105 KDa-band, whereas the cleaved form is visible at 96 KDa. We calculated the fraction of cleaved (inactive) C1-INH in plasma samples from patients with COVID-19 belonging to the different categories. We observed a significant increase in the relative level of cleaved C1-INH in patients compared to controls. In mild-intermediate patients, an up to 2.5-fold increase was observed, whereas in critically ill patients, the ratio decreased (Figure 2b).

### 3.3. Interaction of PKa and C1-INH in COVID-19

To better characterize the activity of C1-INH, we evaluated the amount of PKa:C1-INH complexes in the plasma of COVID-19 patients.

**Figure 3:**
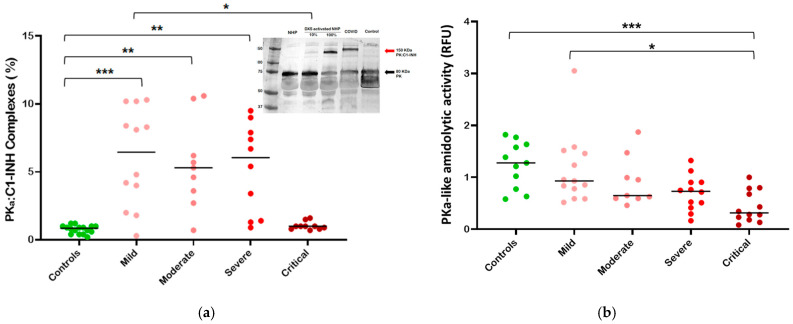
Kallikrein/kinin system analysis in relation to the severity of COVID-19. Dots plot showing (**a**) the plasma levels of PKa:C1-INH complexes and (**b**) the residual activity of kallikrein in plasma. Median (horizontal bars) depict non-Gaussian data; *p* values are from the Mann–Whitney test. Statistical significance is represented with * if *p* < 0.05, ** if *p* < 0.01, *** if *p* < 0.001. The insert displays a Western blot showing the appearance of a high molecular weight band corresponding to plasma PKa:C1-INH complexes in COVID-19 patients and in normal human plasma (NHP) activated with different amounts of dextran sulfate (DXS).

Once more, we found that the quantity of PKa:C1-INH complexes was significantly higher when considering patients with mild to intermediate severity disease compared to healthy controls. No statistically significant differences emerged between controls and critically ill patients (Figure 3a).

Consequently, it can be concluded that in patients with mild to intermediate clinical severity, where an increase in PKa:C1-INH complexes is observed, there is a systemic activation of the kallikrein-kinin system. In contrast, this is not observed in critically ill patients.

Complexes were also visualized by Western blot; in patients with higher levels of complexes, a band around 150 KDa is visible after probing with an anti-PKa antibody (insert of Figure 3a).

In order to evaluate the amount of plasma kallikrein, we also investigated kallikrein spontaneous amidolytic activity. The results demonstrated a significant reduction in spontaneous kallikrein activity in the plasma of critically ill patients (*p* < 0.001) and a slight difference persisting between mild and critical patients (*p* < 0.1) (Figure 3b). This result indicates a progressive consumption of kallikrein.

## 4. Discussion

It has been ascertained that the immunothrombotic proteolytic cascades operating in blood plasma have pivotal roles within the SARS-CoV-2 infection and COVID-19 pathomechanism in both protecting against infection and exacerbating the disease. Hyper-activation contributes to disease severity. As for hyperinflammation, in which CS and CAS appear to play a role, as well as for microvascular endothelial cell injury and pulmonary angioedema, in which the KKS is deeply involved. Indeed, all these systems are highly interconnected, and it is somewhat unreliable to draw a demarcation line differentiating their single contribution [51,52].

C1-INH is an abundant plasma serpin that plays a pivotal role in regulating these systems by inhibiting a number of serine proteases. In addition to inhibiting the CS classical and lectin pathways, C1-INH is a primary inhibitor of FXIIa and PKa. Furthermore, it has inhibitory activity towards plasmin, tissue-type plasminogen activator, activated factor XI, and thrombin [53,54].

Consequently, in the context of COVID-19, C1-INH might mitigate uncontrolled CS activation and collateral lung damage, reduce capillary leakage and subsequent pulmonary edema, inhibit the generation of microthrombi via the KKS and CAS pathways, and preserve the regulatory role of endothelial cells. On the one hand, C1-INH could be directly involved in the pathophysiology of SARS-CoV-2 infection, while, on the other, its use as a drug could offer therapeutic benefits [33,55]. However, to date only a few clinical and preclinical studies have investigated this research field, with inconsistent results [33,38,56].

To interpret the results and better evaluate a possible interplay between C1-INH levels and COVID-19 pathogenesis, it is important to have a clear understanding of the mechanism of action of C1-INH. As already mentioned, C1-INH is a serpin, a family of proteins that uses a unique “molecular mousetrap” mechanism to inhibit target proteases. In its active form, it presents a reactive center loop (RCL) as “bait” for the protease. Upon interaction, the protease cleaves the RCL, triggering a major conformational change in C1-INH. At this point, the reaction follows two potential pathways: one leads to protease inhibition, and the other to proteolysis, resulting in the release of an inactive serpin. Several factors, such as temperature, pH, and cofactor compound availability, which are strictly connected to clinical conditions, can influence which pathway dominates, consequently affecting the efficiency of inhibition. Indeed, even if in excess compared to physiological conditions, C1-INH could be easily overwhelmed by the proteases activated in COVID-19. For these reasons, in addition to measuring total C1-INH level, it is also fundamental to evaluate C1-INH functional levels to estimate C1-INH consumption.

In our study, we observed an increase in plasma concentrations of C1-INH, both total and functional, in all COVID-19 patients (Figure 1). This is consistent with C1-INH being an acute phase protease and mostly confirms transcriptomic and proteomic studies from the literature [25,29,57]. In particular, a direct correlation was observed between the increase in C1-INH levels, especially functional levels, and the WHO classification of COVID-19 severity (Figure 1b).

Notably, a significant reduction in the ratio of C1-INH function (Fx%) to total (tot%) is observed in patients of mild and intermediate clinical severity, but not in those who are critically ill (Figure 2). This signifies that, except for the critical stages of the disease, C1-INH is actively employed to regulate the activation of both target and potentially non-target proteases. Accordingly, in our cohort, the activation of the CAS/KKS is detected in the mild and intermediate categories of clinical severity, wherein we observe an increase in PKa:C1-INH complexes, followed by a return to normal levels in critically ill patients (Figure 3a). The data on PKa:C1-INH complexes are controversial in the literature, with some studies indicating a decrease [57] and others an increase [58] when measured in critically ill patients, whereas when measured in all COVID-19 patients, without extrapolating the class of critics, it is always increased compared to normal controls [59,60].

At the same time, we observed a reduction in PKa activity in COVID-19 patients, directly correlated to disease severity. In fact, this phenomenon was particularly evident in patients with severe disease. This finding is consistent with other studies where a decrease in PK is observed, especially in critically ill patients [59,61]. Conversely, other studies have reported an increase in PKa-like activity [38,62].

Taken together, our data suggest that CAS/KKS is activated in mild and severe disease, prompting an increase in C1-INH to counterbalance this activation. As the disease severity rises, C1-INH intervenes by blocking the PKa, as evidenced by a decrease in PKa activity, an increase in complexes, and an increase in C1-INH degradation.

In critically ill patients, C1-INH levels remain elevated, but the functional-to-total C1-INH ratio (Fx/tot) suggests there is no C1-INH consumption. Additionally, PKa:C1-INH complexes levels are close to those of normal controls, and PKa-like activity is reduced. Taken together, these findings suggest an exhaustion of CAS/KKS.

Since the consumption of C1-INH and the activation of the KKS were observed in non-critical patients, the results of this work support C1-INH supplementation in the early phases of the disease before progression to a critical state.

In line with our conclusion, subcutaneous C1-INH and icatibant regular use were associated with a significantly reduced rate of reported COVID-19 in HAE patients [63].

On the other hand, clinical trials testing C1-INH supplementation in addition to standard of care (SOC) in patients with non-critical SARS-CoV-2 pneumonia showed no clinical advantage in preventing COVID-19 progression when using a recombinant human C1 inhibitor (ConA) [38]. Several factors may account for these results. ConA administration may have occurred too late in the COVID-19 disease course, or the dosing regimen may have been insufficient to inhibit the activated systems adequately and for a prolonged period.

Despite significant progress in controlling the pandemic through a global scientific and medical effort, SARS-CoV-2 continues to cause acute infections and post-acute syndrome.

Indeed, clinical observations made in COVID-19 patients reveal the impact of SARS-CoV-2 infection on the vasculature and detail how the virus promotes (chronic) vascular inflammation contributing to the long-term consequences of COVID-19, also known as “Long COVID syndrome” [64,65,66].

Actually, the circumstances surrounding long COVID remain unclear. One hypothesis is that a dormant SARS-CoV-2 infection of endothelial cells leads to low-level expression and release of COVID-19 proteins on the cell surface, thereby perpetuating intermittent CS [67] and possibly KKS activation. Indeed, C1-INH exhibits a high degree of interaction with the endothelium [22,68], suggesting its possible involvement in the pathogenesis.

Baillie et al. observed that markers of the classical, alternative, and terminal CS were markedly elevated in patients with long COVID. Furthermore, the most predictive single biomarker of long COVID was C1-INH [69]. It is possible that patients in whom C1-INH remains elevated may be more prone to suffer for long COVID. However, the role of C1-INH in long COVID syndrome has yet to be examined in detail. A comprehensive analysis of both the total and functional levels of C1-INH, as the one performed in this study, may prove beneficial in enhancing the characterization of this syndrome.

## Figures and Tables

**Table 1:** COVID-19 severity classification.

Mild	Symptomatic patients without evidence of viral pneumonia or hypoxia.
Moderate	Pneumonia: clinical signs of pneumonia (fever, cough, dyspnea, fast breathing) but no signs of severe pneumonia, including SpO2 ≥ 90% on ambient air).
Severe	Severe Pneumonia: with clinical signs of pneumonia (fever, cough, dyspnea) plus one of the following: respiratory rate > 30 breaths/min, severe respiratory distress, or SpO2 < 90% on ambient air.
Critical	Acute respiratory distress syndrome (ARDS) Sepsis Septic Shock Acute thrombosis

**Table 2:** Antibodies used and conditions.

	Primary Antibody	Secondary Antibody
C1-INH	in-house chicken anti-C1-INH antibody 1:10,000 (0.08 μg/mL)	anti-chicken IgY AP (A9171, Merck) 1:30,000
	1 h RT	1 h RT
PKa:C1-INH complexes	sheep anti-human Prekallikrein antibody (CL20090A Cedarlane) 1:1000	anti-sheep IgG AP (A5187, Merck) 1:30,000
	o.n. 4 °C	1 h RT

**Table 3:** Clinical characteristics of patients, severity classification at the time of sample collection.

Variables	Total	Mild	Moderate	Severe	Critical
Number of patients	45	12	9	12	12
Male, N (%)	24 (53)	7 (58)	4 (44)	7 (58)	6 (50)
Mean age ± SD	58.9 ± 15	52 ± 11.5	65 ± 16	66 ± 17	55.6 ± 11.8
Days of hospitalization (median, IQR)	15 (11–23)	9 (6–14)	17 (15–21)	18 (11–25)	28 (14–62)
Days between onset and sampling (median, IQR)	11 (8–15)	10.5 (7–12)	10 (6–15)	9.5 (8–12)	14.5 (9–24)
Patients with comorbidities, N (%)	38 (84)	8 (66)	9 (100)	11 (91)	10 (83)
Total comorbidities (median, range)	2 (0–5)	2 (0–4)	3 (1–5)	1 (0–5)	1 (0–3)
Obesity, N (%)	16 (35)	4 (33)	4 (44)	3 (25)	4 (33)
Diabetes, N (%)	9 (20)	2 (25)	2 (22)	3 (27)	2 (20)
Hypertension, N (%)	20 (44)	3 (42)	5 (55)	7 (63)	5 (50)
CAD, N (%)	4 (8)	0 (0)	1 (11)	2 (16)	0 (0)
COPD or asthma, N (%)	9 (20)	3 (25)	3 (33)	1 (8)	2 (16)
Chronic nephropathy, N (%)	2 (4)	0 (0)	1 (2)	1 (2)	0 (0)
Cancer, N (%)	2 (4)	0 (0)	1 (11)	0 (0)	1 (8)
Other comorbidities, N (%)	11 (24)	5 (11)	6 (13)	2 (4)	3 (6)
Fever, N (%)	37 (82)	11 (91)	9 (100)	11 (91)	6 (50)
Cough, N (%)	19 (42)	8 (66)	3 (33)	3 (25)	5 (41)
Dyspnea, N (%)	19 (42)	3 (25)	2 (22)	8 (66)	6 (50)
Diarrhea, N (%)	10 (22)	4 (33)	1 (11)	2 (16)	3 (25)
Pneumonia, N (%)	43 (95)	11 (91)	8 (88)	12 (100)	12 (100)
Respiratory failure and invasive ventilation in ICU, N (%)	12 (26)	0 (0)	0 (0)	0 (0)	12 (100)
Sepsis, N (%)	14 (31)	0 (0)	1 (11)	5 (41)	8 (66)
Thromboembolic complications, N (%)	1 (2)	0 (0)	0 (0)	1 (8)	0 (0)
Acute kidney injury, N (%)	3 (6)	0 (0)	0 (0)	0 (0)	3 (25)
Other complications, N (%)	3 (6)	0 (0)	1 (11)	0 (0)	2 (16)
Deaths, N (%)	8 (17)	0 (0)	0 (0)	2 (16)	6 (50)

IQR, interquartile range; CAD, coronary artery disease; COPD, chronic obstructive pulmonary disease; ICU, intensive care unit. Other comorbidities include dementia, psychiatric disorders, multiple sclerosis, and epilepsy.

**Table 4:** Correlation between WHO severity class and markers of inflammation and fibrinolysis. (Spearman correlation coefficients and *p*-values are indicated).

	**CRP**	**IL-6**	**Ferritin**	**D-Dimers**	**Fibrinogen**	**PT**	**INR**	**aPTT**	**Creatinine**
Spearman r	0.4278	0.661	0.4997	0.6821	0.2732	0.1827	0.1528	−0.04082	−0.07626
*p* value	0.0034	<0.0001	0.0022	<0.0001	0.0924	0.2722	0.3598	0.8104	0.6185
	**	****	**	****	ns	ns	ns	ns	ns
	**Neutrophils**		**Lymphocytes**		**Eosinophils**		**Basophils**		**Platelets**
Spearman r	0.6753		−0.5151		0.007591		0.3102		0.0189
*p* value	<0.0001		0.0003		0.9605		0.0404		0.9019
	****		***		ns		*		ns

Statistical significance is represented with * if *p* < 0.05, ** if *p* < 0.01, *** if *p* < 0.001, and **** if *p* < 0.0001. ns: not significant.

## Data Availability

The data presented in this study are available on motivated request from the corresponding author. The data are not publicly available due to privacy restrictions.
